# Insights from the Metagenome of an Acid Salt Lake: The Role of Biology in an Extreme Depositional Environment

**DOI:** 10.1371/journal.pone.0122869

**Published:** 2015-04-29

**Authors:** Sarah Stewart Johnson, Marc Gerard Chevrette, Bethany L. Ehlmann, Kathleen Counter Benison

**Affiliations:** 1 Science, Technology, and International Affairs, Georgetown University, Washington, District of Columbia, United States of America; 2 Graduate Program in Biotechnology and Bioengineering, Harvard University Extension, Cambridge, Massachusetts, United States of America; 3 Division of Geological and Planetary Sciences, California Institute of Technology, Pasadena, California, United States of America; 4 Jet Propulsion Laboratory, California Institute of Technology, Pasadena, California, United States of America; 5 Department of Geology and Geography, West Virginia University, Morgantown, West Virginia, United States of America; Belgian Nuclear Research Centre SCK•CEN, BELGIUM

## Abstract

The extremely acidic brine lakes of the Yilgarn Craton of Western Australia are home to some of the most biologically challenging waters on Earth. In this study, we employed metagenomic shotgun sequencing to generate a microbial profile of the depositional environment associated with the sulfur-rich sediments of one such lake. Of the 1.5 M high-quality reads generated, 0.25 M were mapped to protein features, which in turn provide new insights into the metabolic function of this community. In particular, 45 diverse genes associated with sulfur metabolism were identified, the majority of which were linked to either the conversion of sulfate to adenylylsulfate and the subsequent production of sulfide from sulfite or the oxidation of sulfide, elemental sulfur, and thiosulfate via the sulfur oxidation (Sox) system. This is the first metagenomic study of an acidic, hypersaline depositional environment, and we present evidence for a surprisingly high level of microbial diversity. Our findings also illuminate the possibility that we may be meaningfully underestimating the effects of biology on the chemistry of these sulfur-rich sediments, thereby influencing our understanding of past geobiological conditions that may have been present on Earth as well as early Mars.

## Introduction

Acidic brine lakes are not common, and those that occur naturally are relatively new to scientific study [1, 2]. While the microbial communities in saline environments have been characterized many times [3–7], as have those in environments associated with acidic hot springs and acid mine drainage [8–11], much work remains to be done in understanding the microbial communities in acid salt lakes, where organisms must contend not only with tremendous salinity but also with extreme proton pressure [12, 13]. In addition, acid salt lakes are often associated with complex geochemistry, water stress due to desiccation, dramatic diurnal temperature changes, and high levels of solar radiation. These environments are of particular interest as a source of novel genetic diversity and the understanding of life in conditions of relevance to Earth’s past. For instance, evidence of ancient and widespread acid salt lake and groundwater systems has been recently uncovered over much of the North American mid-continent [14, 15]. These discoveries have prompted a reevaluation of how past surface conditions can be reconstructed along with further investigations into the geochemistry and ecology of acid saline systems in general [14, 16].

**Fig 1:**
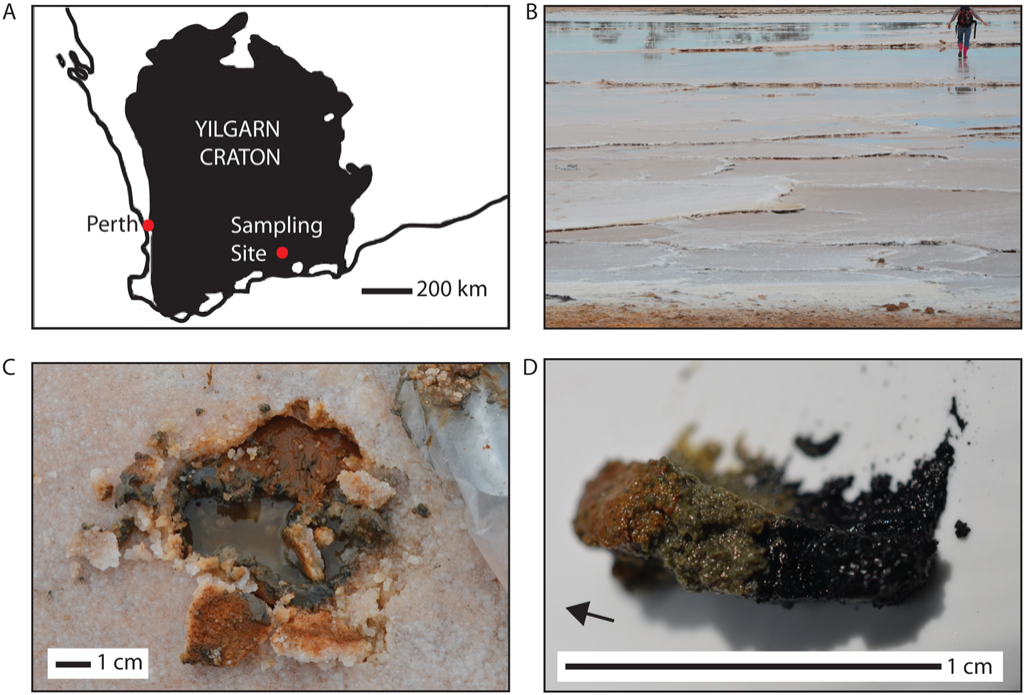
Sampling context. (a) A map of the Yilgarn Craton in Western Australia showing the location of the acid salt lake sampled. (b) At the time of sampling, the acid salt lake was at a stage of evapoconcentration with a maximum depth of 20 cm. (c) Bedding plane of the sampling site, below a 5mm efflorescent halite crust. (d) Within the bulk sediment sample, three distinctive layers were subsampled for VNIR spectroscopy and SEM analysis (orange, green, and black). Arrow indicates stratigraphic up.

One of the few places that acid salt lakes occur naturally is within the Yilgarn Craton of Western Australia. The craton is comprised primarily of deeply weathered Achaean rocks, with red siliciclastic and reworked chemical sediment hosting ephemeral saline lakes [1, 17, 18]. The host rocks include granites, granodiorites, gneisses, anorthosites, quartzites, and ironstones along with some mafic and ultramafic rocks which are present as greenstone belts [1]. Amid this Precambrian bedrock, a regional acid brine groundwater system supplies water to dozens of shallow and ephemeral lakes [18]. The lakes range from several square meters to several square kilometers in size with pHs as low as 1.5 and salinities as high as 32% of total dissolved solids [1]. Here we focus on the physical and biological characteristics of one such inland lake in the Yilgarn Craton, located at 33° 25.567 S, 121° 41.343 E, just east of Grass Patch, Western Australia (Fig 1). Like others in the region, it is marked by abundant aluminum phyllosilicates, chloride, sulfate (including alunite) and iron oxide minerals as well as an absence of carbonates [18, 19]. The lake sediment provides an intriguing matrix of nutrients and solid surfaces for microbial growth. The silicate sediments contain quartz and a variety of clays, and cements coating the silicates include variable iron oxides, chloride, and calcium and magnesium sulfates, as described further herein. Previous geochemical measurements on lake water and groundwater at this lake indicate high levels of dissolved major ions and elements (up to 28,900 mg/L sodium, up to 2720 mg/L magnesium, up to 42,300 mg/L chlorine, up to 5000 mg/L sulfate, up to 399 mg/L potassium, up to 436 mg/L calcium, up to 136 mg/L bromide, up to 220 mg/L silica, up to 331 mg/L aluminum, up to 42 mg/L iron, and an undetectable level of bicarbonate) [20]. In 2005 and 2006, the lake water pH was recorded at 2.7 and 3.3 [20]; field measurements indicated that the lake water pH was 3.6 at the time of our sample collection, similar to field measurements taken in 2001.

While many studies of extreme environments have focused on 16S rRNA gene analysis, advances in sequencing technology, metagenomic approaches, and analytical tools provide a more detailed look into the environmental gene pool and potential functions of biogeochemical relevance within a microbial community. While metagenomics has its limitations—laboratory-based experiments can provide more definitive information on the relative importance of specific genes and organisms in an environment—the approach nevertheless illuminates the functional coding potential of microbes within an ecosystem, especially important in light of the fact that the vast majority of prokaryotes are thought to be uncultivable in the laboratory [21]. Here we use high-throughput DNA sequencing and analysis to examine the taxonomic structure of the microbial community of this acid salt lake as well as its metabolic potential. In order to provide a robust geologic context for the energy harvesting and cell building pathways available to microorganisms living within the sediment matrix, we also analyze the mineralogy of siliciclastic sediments with visible/near-infrared (VNIR) spectroscopy, x-ray diffraction (XRD), and scanning electron microscopy (SEM).

## Materials and Methods

### Ethics statement

No endangered or protected species were involved in this investigation. Field studies permissions were obtained from the Geological Survey of Western Australia (First Floor Mineral House, 100 Plain Street, East Perth WA 6004), and samples collected complied with all provisions of the Australian Commonwealth Government Protection of Movable Cultural Heritage Act (1909). The importation of rocks and sediment into the United States was permitted under the United States Department of Agriculture’s APHIS guidelines (Permit Number P330-10-00014).

### Sample collection

Sediment samples were collected from saline sandflat/mudflat facies flanking the lake during the austral fall when the lake was in an evapoconcentration stage. The lake water was approximately 20 cm deep at maximum depth, with pH and salinity measured at 3.6 and 46 g/l respectively. The lake is fed by a combination of acid saline groundwater and meteoric waters [20], and although the shallow groundwater was not specifically measured, the pH and salinity of the groundwater have been shown in previous studies to be similar to that of the lake water in the evapoconcentration stage [1, 20]. At the time of sampling, much of the lakebed was covered by millimeter-scale efflorescent evaporite halite crystals, forming a crust ranging from a few millimeters to several centimeters in depth that was broken into polygonal expansion ridges. The sediment we sampled consisted of sand and mud-sized grains located below approximately five millimeters of subaerial halite crust at the groundwater table interface (Fig 1). The sediment was split into a bulk sample for biological and XRD analysis and into three discrete subsamples of layers whose colors varied over a depth of approximately one centimeter (an orange sample, a green sample, and a black sample) for VNIR spectroscopy and SEM analysis. The sediment for biological analysis was collected in Nasco (Fort Atkinson, WI, USA) whirl-pak bags using sterile techniques. It was frozen immediately upon collection and during transit to the United States, and then promptly transferred to a -80°C laboratory freezer.

### Mineralogy

The physical properties and chemical composition of the sediment were determined using a combination of techniques. VNIR spectroscopy using reflected light from 0.4–2.5 μm was used to assess subsample mineralogy by diagnostic absorptions in spectra [22]. Samples were measured for reconnaissance in the field and then remeasured in the laboratory relative to a spectralon reflectance standard using an Analytical Spectral Devices (ASD) (Boulder, CO, USA) FieldSpec3 with contact probe attachment. XRD of the bulk sample was used to determine both quantitative bulk mineralogy and clay mineralogy. Analyses were performed under contract with K-T Geoservices Company (Gunnison, CO, USA) for both the bulk sample and the fine (<4 μm) fraction. Ethylene glycol treatment was used to search for the presence of swelling clays. Bulk mineralogy of crystalline and non-swelling phases was estimated using Rietveld refinement. Finally, a 1550 VP Field Emission SEM from Zeiss (Oberkochen, Germany) equipped with an X-Max SDD X-ray Energy Dispersive Spectrometer system from Oxford Instruments (Abingdon, United Kingdom) was used, operating the beam at 10 kV, to study micron-scale textures along with the chemistry of the three subsamples.

### DNA extraction and sequencing library construction

DNA was extracted in triplicate from the sediment using a modified PowerLyzer PowerSoil protocol for DNA from low biomass soil from MO BIO Laboratories (Carlsbad, CA, USA). Throughout the DNA extraction, preparation, and sequencing processes, a negative control without DNA was employed to monitor for contamination. Starting with the dry glass bead tube from the PowerLyzer PowerSoil Kit from MO BIO Laboratories (Carlsbad, CA, USA), 0.25 g of sediment was added to 500 μl of Bead Solution and 200 μl of phenol:chloroform:isoamyl alcohol pH 7–8 from Amresco (Solon, OH, USA). Next, 60 μl of Solution C1 was added, vortexed, and centrifuged at one minute full speed to obtain a pellet. The supernatant was removed and added to the new tube and combined with 100 μl of Solution C2. Then 100 μl of Solution C3 was added and mixed, then incubated at 4°C for 5 minutes. The mixture was centrifuged at one minute full speed to obtain a pellet. The supernatant (~650 μl) was transferred to a new tube and combined with 650 μl of Solution C4 and 650 μl of 100% ethanol. The lysate was loaded 650 μl at a time and bound to the spin column in three steps, alternating with centrifugation. The membrane was then washed with 650 μl of 100% ethanol and then 500 μl of Solution C5. The spin column was centrifuged at two minutes full speed, then transferred to a clean tube. The DNA was eluted in 60 μl of Solution C6.

The DNA was quantified using a Qubit dsDNA High Sensitivity Assay from Life Technologies (Carlsbad, CA, USA). 130 μl of DNA was sheared to a size of 300–500 bp in a Covaris Inc. (Woburn, MA, USA) microtube using a Covaris Inc. (Woburn, MA, USA) LE220 instrument (fill level: 10; duty cycle: 15, PIP: 500, cycles/burst: 200, time: 58 sec). A solid-phase reversible immobilization (SPRI) was performed using AMPure XP beads from Beckman Coulter (Brea, CA, USA) with a 1x volumetric ratio [23]. After ethanol washes, DNA was end repaired “with-bead” with a 100 μl master mix including 88 μl of 1x T4 DNA ligase buffer from New England Biolabs (NEB) (Ipswich, MA, USA), 2 μl of 25 mM dNTP mix, 5 μl of 10 U/μl T4 PNK (NEB), 4 μl of 3 U/μl T4 DNA polymerase I (NEB), and 1 μl of 5 U/μl Klenow fragment of DNA polymerase I (NEB). The DNA library/bead suspension was incubated at room temperature for 30 minutes. Again, a 1x SPRI was performed with a “with-bead” A-tailing 100 μl master mix including 90 μl of 1x NEBuffer 2 (NEB), 5 μl of 10 mM dATP, and 5 μl of 5 U/μl Exo-Minus Klenow DNA Polymerase (NEB) and left to incubate at 37°C for 30 minutes. A 1x SPRI cleanup was performed with an elution in 100 uL of 1x NEB ligation buffer (NEB). Next, 2 μl of NEB DNA Quick Ligase (NEB) and 3 μl of indexed DNA adapters (Illumina, San Diego, CA, USA) were added and mixed thoroughly. The mixture was incubated at room temperature for 15 minutes. Finally, a 1.0x SPRI was again performed with an elution in 50 μL of 1x Tris buffer (10 mM Tris-Cl, pH 8).

The library was amplified using a TruSeq DNA HT Sample Preparation Kit (Illumina, San Diego, CA USA) following the TruSeq DNA Sample Preparation Guide (#15026486 Rev. C, July 2012). PCR conditions were as follows: denaturation at 98°C for 30 sec; 10 cycles of 98°C for 10 sec, 60°C for 30 sec, and 72°C for 30 sec; and final extension at 72°C for 5 min. After amplification was complete, a 1:5 dilution of DNA was run on a 2% agarose gel to verify that the amplification was successful.

A 0.5x volume of AMPure XP beads (Beckman Coulter, Brea, CA, USA) were incubated with the sample to selectively bind fragments >~600 bp. A 0.13x SPRI using 40 μL of fresh beads was performed on the previous supernatant to remove the excess adapter with an elution of 25 μL of 1x Tris buffer. After incubation at room temperature for 5 minutes and separation using a magnet, the solution—the final DNA library, ready for sequencing—was transferred to a fresh 1.5 ml tube.

### Sequencing

The DNA library was sequenced on an Illumina (San Diego, CA, USA) MiSeq platform at the Broad Institute in Cambridge, Massachusetts using a MiSeq Reagent Kit 300 v2 (Illumina, San Diego, CA, USA) to generate paired-end, 2x100 bp reads. The sequences are publicly available on the NCBI server under project ID PRJNA260488.

### Taxonomic characterization

Generated reads were processed via the metAMOS assembly and analysis pipeline [24]. Reads were filtered for quality by metAMOS (1,495,660 out of 1,953,351, or 76.6%, passed QC) and were assembled using SOAPdenovo [25] and mapped by Bowtie2 [26]. Open reading frames were predicted by FragGeneScan [27]. Repeats were identified and consolidated by Repeatoire [28]. The remaining contigs were annotated via BLAST [29], and MetaPhyler [30] was used to assign taxonomic classifiers. Different thresholds were used for each of these parameters, which were automatically learned from the structure of the reference database, reflecting the fact individual bacterial genomes and proteins can have different evolutionary rates, and that metagenomic reads contain gene fragments of different lengths [30]. Scaffolds were created by Bambus2 [31] and functional annotation was again processed using BLAST. Full-length rRNA sequences were used, and chimeric sequences were filtered out as described in [24] during Bambus2 assembly. Taxonomic information was visualized using Krona [32].

### Pathway annotation and analysis

Raw sequencing reads with quality scores lower than 20 were removed by Fastq Groomer and Fastq Quality Trimmer on the GALAXY webserver [33]. Sequences were then error-corrected by Musket [34] and extended by FLASh [35]. Extended and non-extended reads were aligned to the KOBAS Orthology (KO) database [36] (based on the Kyoto Encyclopedia of Genes and Genomes database; KEGG [37]) by BLASTX with an e-value cutoff of 1e-5. Results were analyzed by the KOBAS 2.0 webserver annotation pipeline [36]. These annotations were used to map reads to KEGG pathways and to functionally profile reads mapping to genes involved in sulfur reduction and oxidation by custom perl scripts (freely available at http://github.com/chevrm/kopilot). Pathways were totaled, characterized according to their KEGG functional designation, and visualized as fraction of total reads. For additional genes that have recently been identified as important in sulfur turnover [38], protein sequences were retrieved from the NCBI nr database and built into a protein BLAST database. A 6-frame translated query of reads was queried against this database by BLASTX with an e-value cutoff of 1e-5. Results were searched against the UNIPROT/SWISSPROT [39] database, and top hits were used to confirm search specificity. Confirmed hits were assigned taxon designations via KRAKEN [40].

## Results and Discussion

### Mineralogy

**Fig 2:**
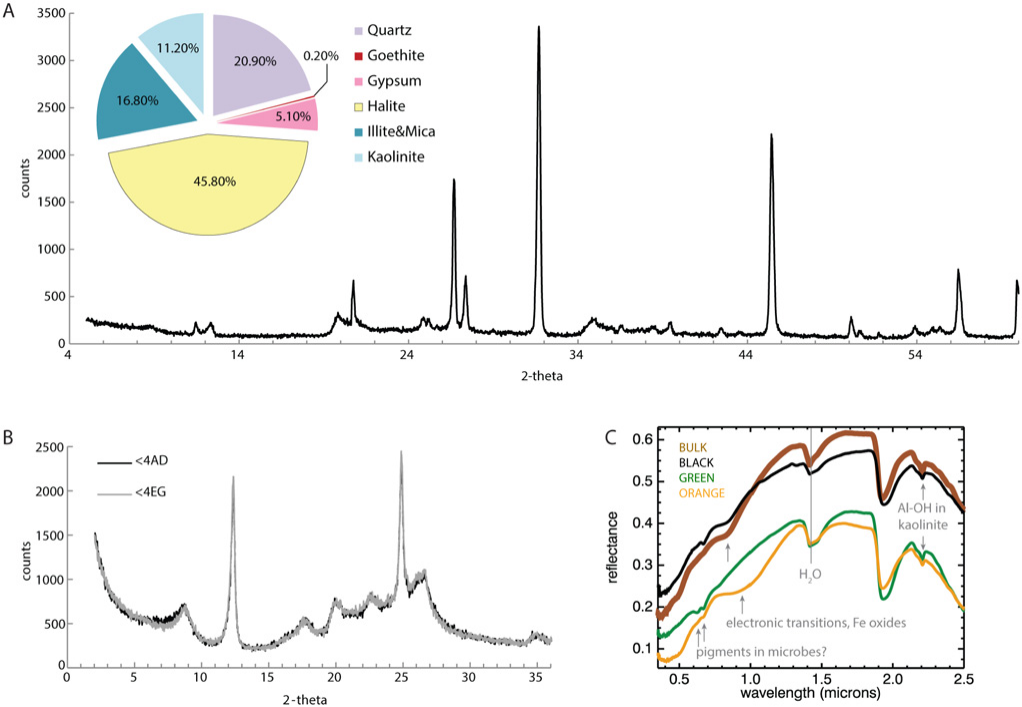
XRD results. (a) XRD pattern from the bulk sediment sample with derived bulk mineralogy is shown in the pie chart. (b) XRD pattern after sieving to the <4 μm fraction showed no change upon ethylene glycol treatment, pointing to an absence of smectite clay minerals. (c) VNIR spectra showed aluminum phyllosilicate in all three discrete layers in the sample with variations in the type and quantity of iron oxides. Small sharp absorptions from 0.6–0.7 μm may be due to pigments in microbes.

**Fig 3:**
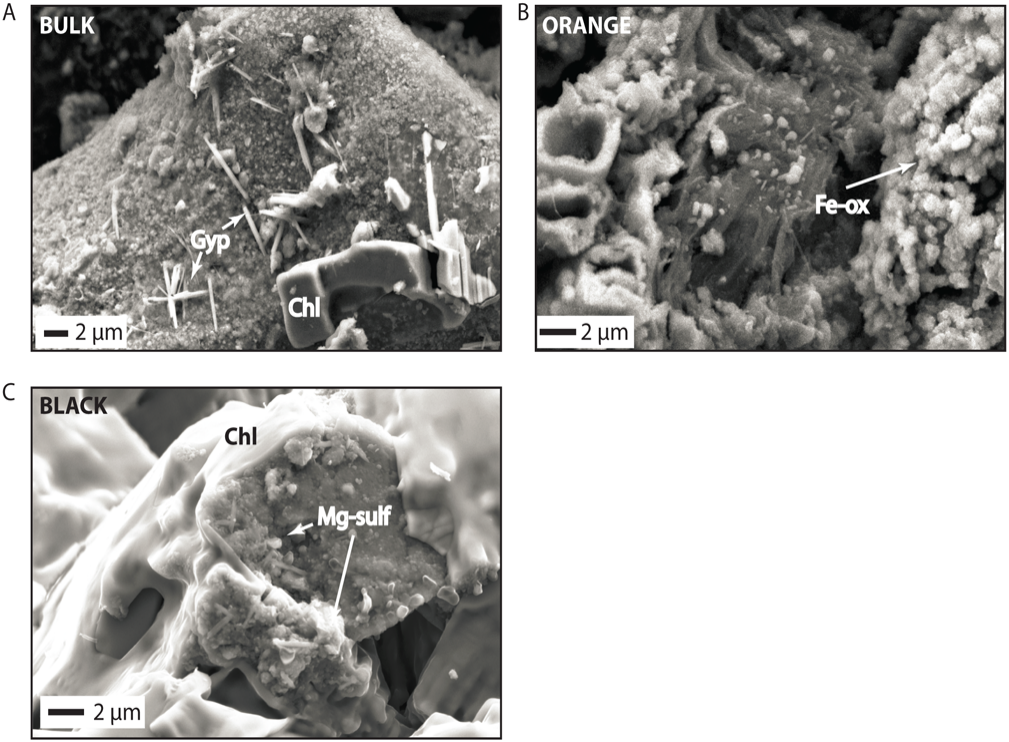
SEM results. (a) Gypsum and chloride precipitates on a silicate substrate (bulk sample). (b) Iron oxide precipitates (orange sample). (c) Magnesium sulfates on a silicate substrate surrounded by precipitated chloride (black sample).

Silicate minerals, which could be detrital or authigenic, comprised half the sediment with the remainder composed of clearly authigenic iron oxides, sulfate salts, and chloride salts (Fig 2). Grain sizes ranged from the hundreds of microns to ~10 microns. Chloride, sulfate, and iron oxide minerals occurred on and around the surfaces of the silicates (Fig 3). SEM analysis suggested some of the silicates could have feldspar-like chemical compositions, but XRD and VNIR spectroscopy analyses instead indicated secondary minerals, such as the phyllosilicates kaolinite and illite. Smectite clays were absent based on a lack of swelling behavior upon ethylene glycol treatment (Fig 2b). Though detected with XRD, no discrete grains of quartz were observed in SEM; these may have been obscured by coatings of precipitated phases.

Iron oxides coated surfaces and also appeared as precipitates (Fig 3b). Interestingly, the composition of the iron oxides appeared to vary by sample. XRD identified goethtite in the bulk sample, though very fine-grained iron oxides often precluded detection by XRD. VNIR spectroscopy showed clear variation in the presence and type of iron oxide between the different subsamples. Absorptions in the bulk sample and black sample were consistent with the presence of hematite, while the orange sample had a hydroxylated iron oxide like goethite, ferihydrite, or lepidocrocite (Fig 2c). Interestingly, there were some small sharp absorptions from 0.6–0.7 μm that did not correspond to absorption features in minerals and may be consistent with pigments in microbes [41]. Approximately half the sediment sample was comprised of evaporite minerals, and sometimes evaporite minerals were observed to entomb all other silicate grains, as with sodium chloride (Fig 3c). Laths of precipitated gypsum, less abundant, were found in all the subsamples. Magnesium sulfates, not detected in XRD, were found in the black sample with SEM but were not observed in other samples. Collectively, phyllosilicates and chlorides were quite similar across samples. Variability in sulfate cation and iron oxide phases may be recording chemical reactions occurring within the upper centimeter of sediment near the sediment-water interface.

### DNA sequencing

**Fig 4:**
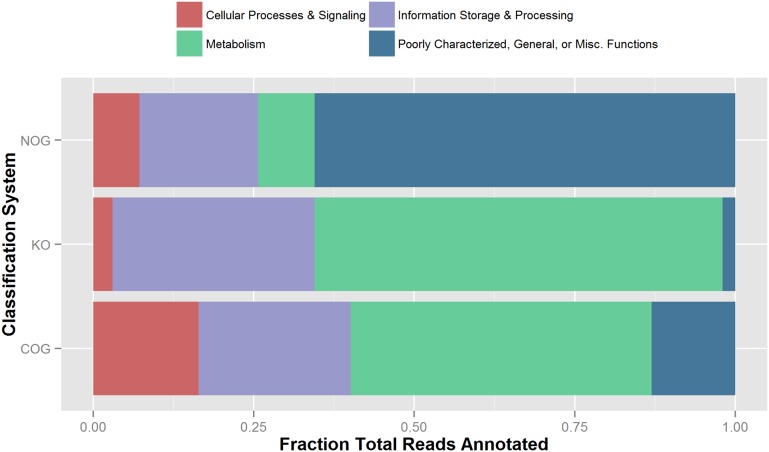
Functional assignment of reads. Groupings are shown for the predominant NOG, KO, and COG identifiers.

The DNA sequencing from the lake’s surface sediment generated 1.9 million sequence reads (201 Mbp) (Table 1). Of these, 255,531 corresponded with alignment-identified protein features, and 45 diverse genes associated with sulfur metabolism were detected. Reads were functionally annotated by MG-RAST to the NOG, KO, and COG functional databases (Fig 4) [36, 42, 43].

**Table 1:** Read statistics.

Parameter	
Base pair count	201,350,619 bp
Total sequence count	1,953,351
Sequences passing QC	1,495,660
Mean sequence length	103 +/- 2 bp
GC percentage	55 +/- 7%
Alignment identified protein features	255,531
Alignment identified rRNA features	1,674
Alignment identified functional categories	218,083

### Taxonomy

Within our dataset, 1,674 rRNA features were identified by mapping to the MG-RAST M5NR database [44]. S1 Fig. shows a rarefaction curve of annotated species richness, a plot of the total number of distinct species annotations as a function of the number of sequences sampled. The alpha diversity of the taxonomic population, a summary of the diversity of organisms in a sample, which can be estimated from the distribution of species-level annotations, was 529 species. The detection of both aerobic and anaerobic species suggests the bulk sample spanned the oxic/anoxic interface.

**Fig 5:**
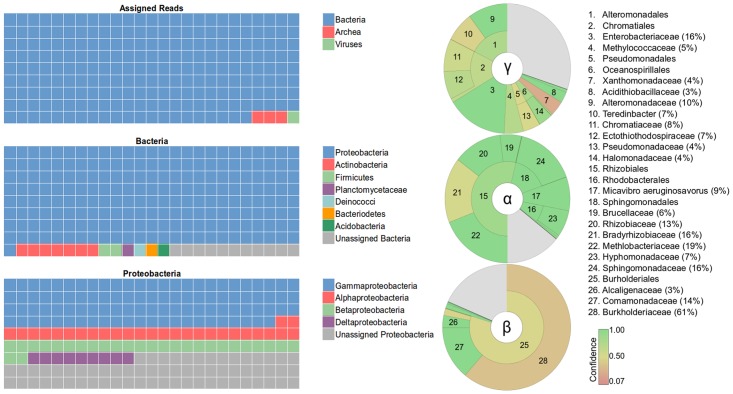
Taxonomic diversity of the sediment. Gray shading indicates the proportion of sequences that could not be assigned to a known phylum, class, or order.

The microbial community of the sediment was dominated by *Gammaproteobacteria* (145,398 reads), followed by *Betaproteobacteria* (40,832 reads), *Alphaproteobacteria* (38,899 reads), *Deltaproteobacteria* (11,978 reads), *Actinobacteria* (9,480 reads), and *Archaea* (7,524 reads), followed by a smaller number of *Firmicutes* (3,327 reads), *Planctomycetes* (2,103 reads), *Deinococcus* (2,008 reads), *Bacteroidetes* (1,890 reads), *Acidobacteria* (178 reads), and *Cyanobacteria* (14 reads) (Fig 5). For both the *Archaea* and *Bacteria*, 5% of the sequences could not be assigned to a known phylum. Of the *Proteobacteria* sequences, 29% could not be assigned to a known class; 30% of *Gammaproteobacteria* sequences, 18% of *Betaproteobacteria* sequences, and 14% of *Alphaproteobacteria* sequences could not be assigned to a known order.

Our taxonomy results suggest the presence of a number of halophiles, and in many cases, they also indicate a potential extension of their known range to include acidic conditions. For instance, *Chromohalobacter salexigens* (4,081 reads), a halophilic and highly halotolerant, gram-negative, aerobic, chemoorganotrophic *Gammaproteobacterium* whose salt requirements can be met by a wide variety of ions, including potassium, rubidium, ammonium, and bromide [45, 46], was abundant within our dataset. *C*. *salexigens* has been shown to have one of the widest salinity ranges for growth found in nature, and it is known for its ability to cope with thermal stress via transcriptional regulation [46–49]. Our results also indicate the presence of *Spiribacter Salinus* (7,132 reads), a newly named aerobic, heterotrophic, halophilic *Gammaproteobacteria* unable to grow in the absence of NaCl [50], as well as *Halorhodospira halophila* (3,032 reads), a recently reclassified halophilic purple bacterium, also within the family *Ectothiorhodospiraceae* and also known to oxidize sulfide to sulfur, which is deposited outside the cell via metabolic pathways that are not yet fully resolved [51, 52]. While other *Ectothiorhodospiraceae* have been found in acidic conditions (including *Acidiferrobacter thiooxidans*, previously known as *Thiobacillus ferrooxidans*, an acidophilic iron-oxidizing bacterium first described 25 years ago [53]), our data suggests that that capacity for acid tolerance may also be held among these recently reclassified organisms, though it is not possible to entirely rule out inputs to the metagenome from the surrounding sandflats, soils, and dunes. Also among the *Proteobacteria*, we detected *Halomonas elongata* (1,151 reads), a wide-ranging halophile that synthesizes and accumulates the compatible solute ectoine to protect proteins from desiccation and temperature extremes [54].

The *Archaea* were dominated by several clades of *Halobacteria*, an extremely halophilic organism that inhabits salt lakes and salterns that can grow on or within salt crystals; while they do not produce chlorophyll like true phototrophs, species of *Halobacteria* contain light-sensitive pigments that can absorb light and trigger ATP synthesis [55]. While most halophilic *Archaea* grow optimally at neutral to slightly alkaline pH, or only at alkaline pH, exceptions are known, including the newly classified *Halarchaeum acidiphilum*, recently isolated from a commercial solar salt [56]. Most abundant in our dataset were the species *Halobacterium salinarum* (561 reads), known for its extreme salt tolerance, bioenergetic flexibility, and presence in mass culture the Great Salt Lake [57, 58], *Halorubrum lacusprofundi* (367 reads), with its adaptations to increase structural flexibility and protein function at low temperature [23], *Halomicrobium mukohataei* (342 reads), of interest for its relatively isolated position in the tree of life [57], and *Haloferax volcanii* (411 reads), the most common microorganism in the sediment of the Dead Sea [59].

In addition to acid and salt tolerance, a number of geochemically relevant species were detected in our data. For instance, *Acidithiobacillus ferrivroans* (3,903 reads) of the class *Acidithiobacillia*, which has recently been proposed to be placed outside of the *Gammaproteobacteria* as a separate lineage, grows by autotrophically utilizing energy derived from the oxidation of elemental sulfur and reduced inorganic sulfur compounds and generates a great deal of sulfuric acid (i.e. hydrogen ions, H^+^, and sulfate ions, SO_4_^-2^) as a product of its metabolism [60]. In addition, among the *Betaproteobacteria* were *Thiomonas intermedia* (718 reads), a moderately acidophilic, gram-negative, aerobic sulfur oxidizer [61], and *Albidiferax ferrireducens* (2,684 reads), of note as a facultative anaerobe whereas most Fe(III) reducing microorganisms are strict anaerobes [62].

The *Deltaproteobacteria* were dominated by *Desulfobulbaceae* (7,988 reads), a family of sulfate-reducing bacteria that consume sulfate in large amounts to obtain energy and expel the resulting sulfide as waste [63]. This is particularly interesting, as to date very few extremely acidophilic sulfur- or sulfate-reducing bacteria have been characterized. It is possible that these organisms grow primarily in consortia, as suggested by a *Desolfosporosinus-*like isolate from the island of Montserrat that has been demonstrated to grow in mixed culture at pH 3.2 and above [64]. Two novel, spore-forming, obligately anaerobic sulfidogens have also been recently isolated, one from a bioreactor at pH 2.4 and most closely related to an uncultured *Desulfitobacterium* [65], and the other from an acidic river, with reported growth as low as pH 3.8, and for which the species name *Desulfosporosinus acididurans* has been proposed [66]. It may be, however, that these organisms are acid-tolerant as opposed to truly acidophilic, growing equally well or better in less acidic environments.

### Sulfur metabolism

We discovered a total of 5,814 reads, mapping to 45 diverse genes, to be linked to sulfur metabolism (Table 2). A large number of genes were associated with the conversion of sulfate to adenylyl sulfate (APS) and the subsequent production of sulfide from sulfite. In this assimilatory pathway, sulfate is initially activated by reaction with ATP to form APS (primarily via sulfate adenylyltransferase, Sat, as well as sulfate adenylyltransferase subunit 1, CysN, and sulfate adenylyltransferase subunit 1, CysD). APS is then converted to 3'-phosphoadenylyl sulfate (PAPS) (via adenylylsulfate kinase, CysC) and reduced to sulfite (via phosphoadenosine phosphosulfate reductase, CysH), and sulfite is further reduced to sulfide by the assimilatory sulfite reductase (CysI, CysJ, and Sir).

**Table 2:** Sequences associated with sulfur metabolism.

Total reads assigned	Unique taxa assigned	Pipeline	KEGG orthology	Description	Gene	Most specific taxon assignment	Clade
1,536	6	BLAST, nr*	K00958	Sulfate adenylyltransferase	*sat*	*Acidithiobacillus*	*Acidithiobacillia*
						*Prokaryotae*	*Unclassified*
						*Halobacteriaceae*	*Halobacteria*
						*Leifsonia*	*Actinobacteria*
						*Sphingobium*	*Alphaproteobacteria*
						*Mycobacterium*	*Actinobacteria*
763	4	BLAST, nr	K00303	Sulfur oxidation protein soxB	*soxB*	*Bacillus*	*Bacilli*
						*Actinomycetes*	*Actinobacteria*
						*Rhizobium*	*Alphaproteobacteria*
						*Burkholderia*	*Betaproteobacteria*
761	7	BLAST, nr	K17230	Fumarate reductase flavoprotein subunit	*fccA*	*Purple photosynthetic bacteria*	*Gammaproteobacteria*
						*Prokaryotae*	*Unclassified*
						*Halobacteriaeaceae*	*Halobacteria*
						*Sphingomonadaceae*	*Alphaproteobacteria*
						*Geodermatophilus*	*Actinobacteria*
						*Rhodothermus*	*Bacteroidetes*
						*Archaea*	*Unclassified*
557	0	BLAST, nr	K17229	Sulfide dehydrogenase [flavocytochrome c]	*fccB*		
554	1	KOBAS	K00381	Sulfite reductase (NADPH) hemoprotein beta-component	*cysI*	*Sphingomonadaceae*	*Alphaproteobacteria*
469	0	KOBAS	K00380	Sulfite reductase (NADPH) flavoprotein alpha-component	*cysJ*		
366	2	BLAST, nr	K17218	Sulfide:quinone oxidoreductase	*sqr*	*Halobacteriaceae*	*Halobacteria*
						*Rhodobacteraceae*	*Alphaproteobacteria*
349	0	KOBAS	K10764	O-succinylhomoserine sulfhydrylase	*metZ*		
338	1	KOBAS	K12339	Cysteine synthase B	*cysM*	*Cenibacterium*	*Betaproteobacteria*
228	0	KOBAS	K01738	Cysteine synthase A	*cysK*		
226	1	KOBAS	K00640	Serine O-acetyltransferase	*cysE*	*Acidithiobacillus*	*Acidithiobacillia*
202	8	KOBAS	K00390	Phosphoadenosine phosphosulfate reductase	*cysH*	*Halorhabdus*	*Halobacteria*
						*Natronomonas*	*Halobacteria*
						*Halomicrobium*	*Halobacteria*
						*Haloferax*	*Halobacteria*
						*Halobacteriaeaceae*	*Halobacteria*
						*Geodermatophilus*	*Actinobacteria*
						*Natrialba*	*Halobacteria*
						*Halopiger*	*Halobacteria*
168	0	BLAST, nr	K01362	Adenylylsulfate reductase membrane anchor	*aprM*		
147	0	KOBAS	K00860	Adenylylsulfate kinase	*cysC*		
132	0	KOBAS	K01082	3'(2'), 5'-bisphosphate nucleotidase	*cysQ*		
103	3	KOBAS	K01011	Thiosulfate/3-mercaptopyruvate sulfurtransferase	*sseA*	*Natrinema*	*Halobacteria*
						*Modestobacter*	*Actinobacteria*
						*Haloterrigena*	*Halobacteria*
89	1	BLAST, nr		Glutamate synthase (NADPH) small subunit	*dsrL*	*Purple photosynthetic bacteria*	*Gammaproteobacteria*
67	0	BLAST, nr	K00394	Adenylylsulfate reductase subunit alpha	*aprA*		
42	0	BLAST, nr	K17225	Sulfite dehydrogenase soxC	*soxC*		
35	0	BLAST, nr	K00395	Adenylylsulfate reductase subunit B	*aprB*		
31	0	BLAST, nr		Sulfur oxidation V protein	*soxV*		
20	0	BLAST, nr		Cytochrome c oxidase subunit II	*soxH*		
19	0	BLAST, nr		Sulfocyanin, blue copper protein	*soxE*		
19	0	BLAST, nr	K16887	Quinone-interacting membrane-bound oxidoreductase complex, subunit C	*qmoC*		
16	0	BLAST, nr		Intracellular sulfur oxidation protein DsrK	*dsrK*		
13	0	KOBAS	K00955	Bifunctional enzyme CysN/CysC	*cysN/C*		
12	0	BLAST, nr		Quinol oxidase-2, Rieske iron-sulfur protein-2	*soxF*		
10	0	KOBAS	K15555	Sulfonate transport system ATP-binding protein	*ssuB*		
10	0	BLAST, nr		Thioredoxin SoxW	*soxW*		
9	0	BLAST, nr	K16885	Quinone-interacting membrane-bound oxidoreductase complex, subunit A	*qmoA*		
8	1	KOBAS	K11180	Sulfite reductase alpha subunit	*dsrA*	*Asakusa*	*Gammaproteobacteria*
8	0	BLAST, nr	K07235	Intracellular sulfur oxidation protein DsrE	*dsrE*		
6	0	BLAST, nr	K11179	Dissimilatory sulfite reductase complex, gamma subunit	*dsrC*		
6	0	BLAST, nr	K16886	Quinone-interacting membrane-bound oxidoreductase complex, subunit B	*qmoB*		
5	0	KOBAS	K00956	Sulfate adenylyltransferase subunit 1	*cysN*		
5	0	BLAST, nr		Protein involved in sulfur oxidation dsrS	*dsrS*		
4	0	KOBAS	K02045	Sulfate transport system ATP-binding protein	*cysA*		
4	0	BLAST, nr		Intracellular sulfur oxidation protein DsrN	*dsrN*		
2	1	KOBAS	K10831	Taurine transport system ATP-binding protein	*tauB*	*Actinoplanes*	*Actinobacteria*
2	0	KOBAS	K02046	Sulfate transport system permease protein	*cysU*		
2	0	KOBAS	K01739	Cystathionine gamma-synthase	*metB*		
2	0	KOBAS	K03119	Taurine dioxygenase	*tauD*		
2	0	KOBAS	K16937	Thiosulfate dehydrogenase [quinone] large subunit	*doxD*		
2	1	BLAST, nr		Intracellular sulfur oxidation protein DsrO	*dsrO*	*Burkholderiales*	*Betaproteobacteria*
1	1	KOBAS	K00392	Sulfite reductase (ferredoxin)	*sir*	*Geodermatophilus*	*Actinobacteria*

* “nr” refers to the NCBI nr database.

Although the sediment nearest to the surface was at least partially aerobic, we nevertheless detected some genes associated with dissimilatory sulfate reduction for which sulfate or sulfur serves as the terminal electron acceptor of the respiratory chain producing inorganic sulfide. In this pathway, APS is directly reduced to sulfite (via adenylylsulfate reductase subunit alpha, AprA, and adenylylsulfate reductase subunit beta, AprB), and sulfite is further reduced to sulfide by the dissimilatory sulfite reductase (DsrA). Since assimilatory sulfate reduction leads to the biosynthesis of sulfur-containing amino acids instead of the direct excretion of sulfide, the paucity of sulfides in our mineralogical results suggests this dissimilatory pathway may be limited. It is interesting to note, however, that some chemolithoautotrophic sulfur oxidizers, such as the *Thiobacillus denitrificans* detected at low levels in our acid salt lake sediment (15 reads), are thought to be capable of utilizing these enzymes in the reverse direction, forming a sulfur oxidation pathway from sulfite to APS and then to sulfate [67]. Sulfur-oxidizing proteins associated with the Sox system, a sulfur oxidation pathway found in both photosynthetic and non-photosynthetic sulfur-oxidizing bacteria, were also found in abundance. The detection of these specific genes linked to processes involved with sulfur oxidation suggests that microbial activity is in fact generating acidity, particularly in the local environment surrounding the microbes, thereby affecting mineral formation and mineral stability fields.

### Implications for understanding past geobiological environments

Although acidic saline depositional environments are rare today, they may have played an important role in Earth history. In 1998, the first ancient acid lake and groundwater system, the Opeche Shale in the Williston Basin, was identified in the rock record; the formation dates to the Permian, covers more than 200,000 km^2^, and is believed to have formed under similar conditions as those present in modern-day Western Australia [14, 68]. Another formation of roughly the same age, the Nippewalla Group of Kansas, has been found to host siliciclastics and evaporites that record the evolution from a perennial freshwater lake system to a ephemeral acid salt lake system [15].

These two geologic units, covering a vast part of the North American mid-continent, have given rise to a new paleogeographic and paleoclimatic model for understanding western Pangaea [15]. Understanding what kind of microbial populations may have inhabited these acidic environments and how they affected nutrient cycling, climate, and the minerals preserved in the geologic record thereby stands to generate a more global view of the evolutionary forces that have shaped life on Earth.

The Western Australian acid salt lakes and associated mudflats, sandflats, dunes, and soils also provide a modern-day analog for understanding the past conditions on Mars. In 2004, the Mars Exploration Rover mission discovered an ancient aqueous sedimentary system at the Meridiani Planum landing site that was characterized by an abundance of sulfur and very high acidity [69]. The finding was made through detection of characteristic minerals that form at extremely low pH, validating hypotheses that had been made over the years about the existence of acid brines on Mars [19, 70, 71]. Subsequent geological, mineralogical, and geochemical orbital data from multiple orbiter missions (e.g. Mars Express, Mars Odyssey, and the Mars Reconnaissance Orbiter) also suggest the presence of acid sulfate salts in diverse areas across the planet’s surface [72–74]. It is now widely believed that some hydrated sulfates on Mars precipitated from acid saline shallow surface waters and/or groundwaters under an arid atmospheric regime [18, 19, 69, 75–77]. Thus our understanding of mineral formation and mineral stability in these depositional environments stands to deepen our understanding of the climatic history and surface processes preserved in the sedimentary rocks on Mars.

Perhaps most tantalizing, however, is the possibility that microbes influenced the acidity of these sediments and/or precipitated characteristic minerals as a byproduct of their metabolism. For example, a type of crystalline jarosite that is stable in the presence of water can be formed by *Acidithiobacillus* in both aerobic and anaerobic environments, and thus its detection on Mars, particularly in the context of persistent aqueous processes, may serve as a biomarker for microbial sulfide oxidation [78]. Our results suggest that microbes adapted to these acidic, saline depositional environments not only form sulfur minerals directly but also can affect the chemistry of the sediments via their metabolism, thereby influencing which minerals are precipitated abiotically. This finding suggests exciting possibilities for future work in elucidating the fine-scale biogeochemical gradients in acid salt lakes as well as investigating the preservation of biological signatures in Mars-like paleolake sediments.

## Conclusions

In this study, we analyzed the taxonomic diversity and metabolic pathways utilized by the microbial community within the depositional environment of an acid salt lake, an extreme site where life has adapted to contend with both high levels of salinity and daunting proton pressure. Within the sediment matrix, we found evidence for a wide array of microbial organisms, including many species that generate sulfuric acid as a product of their metabolism. In addition, we identified 45 diverse sulfur genes within our metagenomic data, primarily linked to the conversion of sulfate to adenylylsulfate and the subsequent production of sulfide from sulfite or the oxidation of sulfide, elemental sulfur, and thiosulfate via the sulfur oxidation (Sox) system. Our results illuminate the possibility that we may be meaningfully underestimating the effects of biology on the chemistry of these sulfur-rich depositional environments, thereby influencing our understanding of acid salt lake paleoenvironments on Earth as well as Mars.

## Supporting Information

S1 Fig Rarefaction curve. (TIF)
