# Supplementary figures and images for: Insights from the Metagenome of an Acid Salt Lake: The Role of Biology in an Extreme Depositional Environment
Source: PLoS One. 2015 Apr 29;10(4):e0122869. doi: 10.1371/journal.pone.0122869 (PMC4414474; doi:10.1371/journal.pone.0122869)

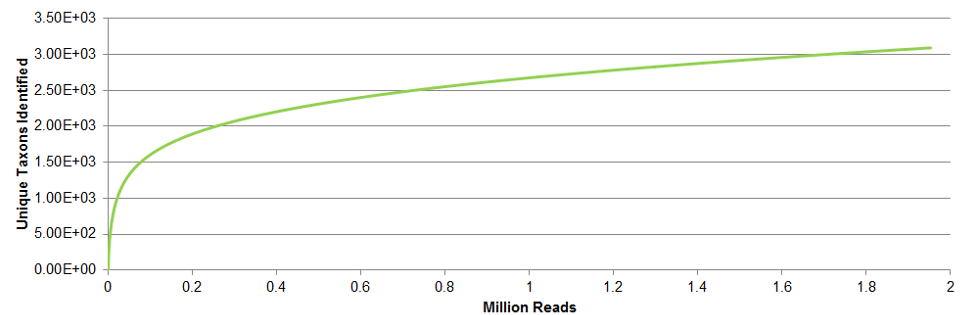

Supplement: S1 Fig — (TIF) [file pone.0122869.s001.tif]
